# Microbial Diversity of the Hell Creek Watershed at the Tri-Faith Community in Nebraska, Based on 16S rRNA Gene Amplicon Sequencing

**DOI:** 10.1128/MRA.00467-20

**Published:** 2020-05-28

**Authors:** John A. Kyndt

**Affiliations:** a College of Science and Technology, Bellevue University, Bellevue, Nebraska, USA; Georgia Institute of Technology

## Abstract

Hell Creek’s watershed is a historically important native land area located in Omaha, Nebraska, that includes Hell Creek and an adjacent flood plain. This initial microbial analysis showed that even though samples were isolated from the same watershed area, there were significant differences between the creek itself and the nearby pond.

## ANNOUNCEMENT

**Fig 1:**
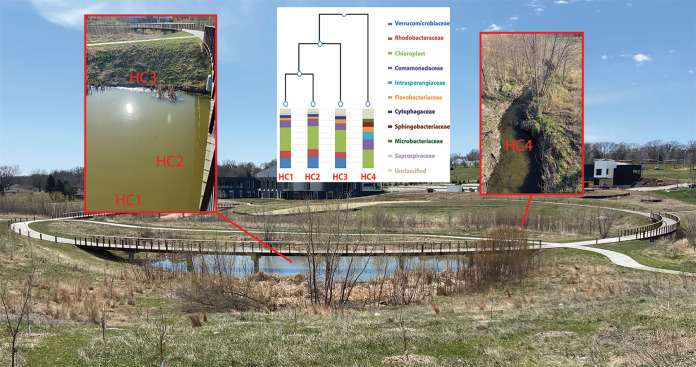
Overview of Hell Creek watershed with sampling locations HC1 to HC4 marked. Samples HC1 to HC3 were taken from the reservoir pond, while HC4 was taken from the creek that runs through the entire area. The center image is a dendrogram showing the hierarchical clustering of samples based on family-level classifications of 16S rRNA gene amplicon samples. Only families with >3% of the sequencing reads are represented.

The Hell Creek watershed area was once an established prairie, savanna, and forest, before development of homes and business roads caught up with the watershed in the past two decades. This significantly reduced the ability to absorb rainwater, making Hell Creek more flood-prone and a possible source of harmful coliform bacteria (1, 2). The Hell Creek area is also the location of the globally unique Tri-Faith Commons (https://www.tenxtenstudio.com/trifaith; 3), and with this new establishment, a restoration of the native watershed area has been started. This includes selected plantings that promote local wildlife and rain gardens that reduce runoff. Hell Creek runs north to south diagonally through the area, which also includes a nearby stand-alone reservoir pond (Fig. 1).

We isolated samples from both the creek and the nearby pond in April 2020. Two pond samples were taken from the center of the pond, ∼6 m apart (HC1 and HC2), while a third pond sample was taken near the edge of the pond (HC3). A fourth sample (HC4) was taken from Hell Creek itself, near the pond (41°14′37.15ʺN, 96°7′2.42ʺW) (Fig. 1). Samples were collected in sterile collection tubes and immediately transferred to the lab, where they were stored at 4°C. The next day, 10 ml of each sample was centrifuged for 15 min at 16,000 × *g* to form a biomass pellet. Total DNA was extracted using the PureLink microbiome DNA purification kit (Invitrogen). Utilizing Qubit and NanoDrop technologies, we determined the quality and quantity of DNA, showing *A*_260_/*A*_280_ ratios of 1.76 (HC3) to 1.94 (HC4). A 16S rRNA amplicon sequencing library was prepared for each sample, following the 16S Metagenomic Sequencing Library Preparation protocol (Illumina; https://support.illumina.com/documents/documentation/chemistry_documentation/16s/16s-metagenomic-library-prep-guide-15044223-b.pdf). Amplicon primers targeting the V3 and V4 region (4) including the Illumina adapter overhang sequences are described in the Illumina library prep protocol and were synthesized by Sigma. The samples were sequenced using a 1.8-pM library with an Illumina MiniSeq instrument. Paired-end (2 × 150 bp) sequencing generated 1,714,184 (HC1), 1,744,434 (HC2), 1,649,932 (HC3), and 1,539,232 (HC4) reads. The primer sequences were removed, and reads with a low quality score (average score, <20) were filtered out using the FASTQ toolkit within BaseSpace version 2.2.0 (Illumina). The 16S Metagenomics application (version 1.0.1) within BaseSpace was used to perform a taxonomic classification, which uses an Illumina-curated version of the GreenGenes taxonomic database and the RDP naive Bayes taxonomic classification algorithm with an accuracy of >98.2% at the species level (5). Paired reads were used for a separate 16S metagenomic analysis for each individual sample. An aggregate comparison and hierarchal clustering of the taxonomic classification results of all 4 samples were also performed within the 16S metagenomic analysis (Fig. 1, insert). Default parameters were used for all software unless otherwise noted.

All samples had more than 88% of the reads identified at the genus level. All three of the pond samples contained abundant representation of the *Verrucomicrobiaceae* (∼15%) and *Rhodobacteraceae* (>10%) families, which were nearly absent from the HC4 creek sample (Fig. 1, insert). In contrast, HC4 contained substantial amounts (∼10%) of *Intrasporangiaceae* (of the *Actinobacteria* phylum [6]), which were found to be less than 1.5% of organisms in the pond samples. All of the samples contained substantial amounts of chloroplast (algal) (∼30%) and *Comamonadaceae* (∼10%), but few to no typical coliform bacteria were identified.

According to a Shannon species diversity analysis (7, 8) run within the 16S Metagenomics application in BaseSpace, each of the samples contained over 2,000 potential species, with substantial differences between the creek and pond water isolates. This establishes a good baseline for further studies of microbiological diversity and ecological impacts of the restoration of this locally important watershed.

### Data availability.

The 16S rRNA gene amplicon data sets have been deposited at DDBJ/ENA/GenBank under BioProject number PRJNA627532 and can be accessed with the BioSample numbers SRS6521435 (HC1), SRS6522831 (HC2), SRS6523000 (HC3), and SRS6523533 (HC4).

## References

[1] Grint A, Laster L 2020 It happened here before. Papio-Missouri River Natural Resources District. Accessed 1 April 2020 https://www.papionrd.org/flood-control/it-happened-here-before/.

[2] Walton K 2017 A river runs around it. UNO Magazine, summer 2017 https://www.unomaha.edu/news/2017/08/river-runs-around-it.php.

[3] Morris F 2015 In America’s heartland, building one home for three faiths. NPR Kios, 17 December 2015 https://www.npr.org/2015/12/17/460149212/in-americas-heartland-building-one-home-for-three-faiths.

[4] Klindworth A, Pruesse E, Schweer T, Peplies J, Quast C, Horn M, Glöckner FO 2013 Evaluation of general 16S ribosomal RNA gene PCR primers for classical and next-generation sequencing-based diversity studies. Nucleic Acids Res 41:e1. doi:10.1093/nar/gks808. 22933715 PMC3592464

[5] Wang Q, Garrity GM, Tiedje JM, Cole JR 2007 Naïve Bayesian classifier for rapid assignment of rRNA sequences into the new bacterial taxonomy. Appl Environ Microbiol 73:5261–5267. doi:10.1128/AEM.00062-07. 17586664 PMC1950982

[6] Stackebrandt E, Scheuner C, Göker M, Schumann P 2014, The family *Intrasporangiaceae* *In* Rosenberg E, DeLong EF, Lory S, Stackebrandt E, Thompson F (ed), The prokaryotes. Springer, Berlin, Germany. doi:10.1007/978-3-642-30138-4_176.

[7] Spellerberg IF, Peter JF 2003 A tribute to Claude Shannon (1916–2001) and a plea for more rigorous use of species richness, species diversity and the “Shannon-Wiener” Index. Global Ecol Biogeogr 12:177–179. doi:10.1046/j.1466-822X.2003.00015.x.

[8] Tuomisto H 2010 A diversity of beta diversities: straightening up a concept gone awry. Part 1. Defining beta diversity as a function of alpha and gamma diversity. Ecography 33:2–22. doi:10.1111/j.1600-0587.2009.05880.x.

